# An Invertible Mathematical Model of Cortical Bone’s Adaptation to Mechanical Loading

**DOI:** 10.1038/s41598-019-42378-5

**Published:** 2019-04-10

**Authors:** Jitendra Prasad, Ajay Goyal

**Affiliations:** Department of Mechanical Engineering, Indian Institute of Technology Ropar, Nangal Road Rupnagar, Punjab, 140001 India

## Abstract

Determination of mechanical loading regimen that would induce a prescribed new bone formation rate and its site-specific distribution, may be desirable to treat some orthopaedic conditions such as bone loss due to muscle disuse, e.g. because of space flight, bed-rest, osteopenia etc. Site-specific new bone formation has been determined earlier experimentally and numerically for a given loading regimen; however these models are mostly non-invertible, which means that they cannot be easily inverted to predict loading parameters for a desired new bone formation. The present work proposes an invertible model of bone remodeling, which can predict loading parameters such as peak strain, or magnitude and direction of periodic forces for a desired or prescribed site-specific mineral apposition rate (MAR), and vice versa. This fast, mathematical model has a potential to be developed into an important aid for orthopaedic surgeons for prescribing exercise or exogenous loading of bone to treat bone-loss due to muscle disuse.

## Introduction

Bone adapts to exogenous mechanical loading^1^. For example, bone in the playing arm of a tennis player may have bone cross-sectional area as much as 35% more than that in the non-playing arm^2^. Conversely, disuse of muscle such as in space-flight can induce hip bone density loss up to 2% per month^3^. Mechanical strain engendered in long bones of various species during normal physical activities is strikingly similar, namely in the range of 0–0.2%, i.e. 0–2000 microstrain (με)^4^. There exists an upper threshold of strain above which new bone formation starts to keep up with the increased mechanical strain^5^. Similarly there is a lower threshold below which bone resorption starts in order to optimize bone mass to the decreased level of mechanical loading^5^. With a plenty of experimental studies it has been established that new bone formation not only depends on the strain magnitude but also on loading waveform, number of cycles, number of bouts and time between the loading bouts^6–9^. Accordingly, there have been efforts to numerically or parametrically relate new bone formation to loading parameters^7,10–12^. The average bone formation rate (BFR) has been explicitly expressed in terms of the loading parameters in some cases^11,12^. Site-specific bone formation has been numerically modeled in other cases^7,10^. There is, however, no consensus regarding how exactly the new bone formation relates to the mechanical environment^10^. Moreover, existing numerical models for site-specific new bone formation are not invertible, which means loading parameters cannot be easily determined for a desired average or site-specific bone formation rate.

The objective of this study is to develop a mathematical model of bone adaptation that can be easily inverted. Most of the bone adaptation models are based on strain magnitude^7,12^, strain energy^10,13^, fluid shear^10,14^ and fatigue^11^ as mechanical stimuli. This work attempts to integrate most of these theories into one. In addition, relevant biological factors such as bone cell network of osteocytes and osteoblasts have also been used to include cell-to-cell communication resulting in intracellular calcium (Ca2+) signaling^15–19^, as implemented by Srinivasan *et al*.^7,20^. It is well established that the molecular cascade that follows a mechanical loading has an important role of Ca2+ as a secondary messenger in activation of the transcription factor NFAT (Nucleus Factor for Activation of T-Cells), which has been implicated in new bone formation through Calcineurin (CaN), CAMK (Ca2+/calmodulin-dependent protein kinase) and MAPK (Mitogen-activated protein kinase)^15^. In the present new model, biological signal transduction is purposefully much simplified in order to maintain invertiblity, and communication between the cells along the cell network has been modeled as diffusion. The model is inherently based on accumulated damage of material due to fatigue loading as a stimulus, and therefore automatically relates stress amplitude and number of loading cycles to new bone formation rate. The effect of fluid flow on new bone formation has been additionally captured through viscoelasticity-like macroscopic behavior of the bone tissue.

Rest of this paper is organized as follows. Section 2 describes the methodology used for computation of overall bone formation rate (BFR) and site-specific mineral apposition rate (MAR). Section 3 details the results, which have been discussed in Section 4. Conclusions have been drawn in Section 5.

## Methods

### The Average BFR Model

**Figure 1:**
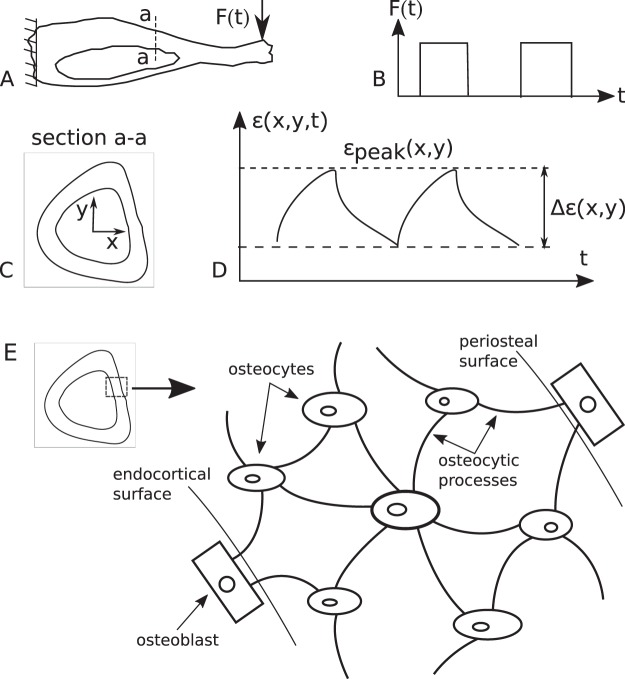
A long bone loaded by a cyclic force, resulting strain at a cross-section of interest and the osteocyte-osteoblast network. (**A**) Tibia fixed at proximal end and loaded at the distal end. (**B**) The periodic load F(t) is composed of a number of cycles of a waveform (e.g. rectangular waveform as shown). (**C**) The idealized mid-diaphyseal cross-section of tibia with its coordinate system. The bone cross-section used for this study is adapted from Srinivasan *et al*.^7^. The outer and inner curves correspond to periosteal and endocortical surfaces, respectively. The section corresponds to the mid-diaphysis of a 16 week old C57BL/6 mouse. Origin of the Cartesian coordinate system (x, y) is at the centroid of the bone section. x and y axes are aligned with lateral and anterior anatomical directions, respectively. (**D**) The strain induced by the cyclic loading has different waveform than the loading waveform due to damping effects. (**E**) The osteocytes, through their processes, are connected to osteoblasts. Strain above a threshold makes an osteocyte release calcium ions (Ca2+) in its cytoplasm. These ions diffuse through the osteocytic processes to reach osteoblasts, which utilize Ca2+ for new bone formation.

*In vivo* experiments involve loading a bone (Fig. 1(A)) with a periodic force *F*(*t*), composed of a number of cycles *N* of a loading waveform (Fig. 1(B)). This loading is repeated for a number of days *d* every week and the loading is typically continued for a number of weeks, end of which bone formation rate (BFR) or mineral apposition rate (MAR) is computed at a bone section (Fig. 1(C)) based on labeling and histological techniques^6,7,21–23^. The loading induces strain field *ε*(*x*, *y*) on the section of interest (Fig. 1(C)). The strain at a point on that section may have a different waveform than the force due to viscoelastic effects, e.g. arising because of fluid flow (Fig. 1(D)). Some *in vivo* experiments are accompanied with strain-gauging and finite element analysis (FEA) to ascertain global peak strain *ε*_peak_ and its location on the section^6,7,21,22^. If *ε*_peak_ is more than a threshold strain *ε*_thres_, new bone formation has been observed in the literature. We hypothesize that bone formation rate for the section in consideration can be given by

1 $$\begin{array}{c}B=p({\varepsilon }_{peak}-{\varepsilon }_{thres})\frac{{\rm{\Delta }}{\varepsilon }_{{\max }}}{{\varepsilon }_{peak}}{N}^{q}{d}^{\beta }{\rm{for}}\,{\varepsilon }_{peak}\ge {\varepsilon }_{thres}\\ \,=0,\,{\rm{otherwise}}.\end{array},$$

where *p*, *q*, ε_thres_ and *β* are the parameters to be determined. Δε_max_ is the amplitude of oscillation of strain at that point (Fig. 1(D)). For a given loading *F*(*t*), both ε_peak_ and Δε_max_ depend on the material properties of the bone, viz. Young’s modulus *E* and effective viscosity *η* arising from the effect of interstitial fluid flow inside bone. *E* for bone may be assumed to be 20 GPa^24^. *η* is the fourth constant to be determined. Using the beam theory and assuming the bone to be a Kelvin-Voigt material, ε_peak_ and Δε_max_ may be easily computed, as given in the Supplementary Methods available online, for any periodic loading. Calcein/alizarin labelling days have not been included in equation (1) assuming BFR remains constant while new bone is being labeled. This assumption may not be valid if labelling days are not appropriately chosen.

Equation (1) has been derived analogous to fatigue damage^11^ following fatigue failure theory^25,26^, (see Supplementary Methods). This equation can be mathematically fit to the BFR data found from an experiment for different loading protocols, numbered *l* = 1 to *n*_*l*_. For each loading protocol, bone formation rate may be computed as follows:

2 $${B}_{l}=p({\varepsilon }_{peak}^{l}(\eta )-{\varepsilon }_{thres}){\xi }_{l}(\eta ){N}_{l}^{q}{d}_{l}^{\beta }$$

where ${\xi }_{l}(\eta )=\frac{{\rm{\Delta }}{\varepsilon }_{{\rm{\max }}}^{l}(\eta )}{{\varepsilon }_{peak}^{l}(\eta )}$, which is a function of viscosity *η*.

Parameters *p*, *ε*_*thres*_, *η*, *q* and *β* can be obtained by minimizing the following error squared:

3 $$f(p,q,\eta ,{\varepsilon }_{thres},\beta )={\sum }_{l=1}^{{n}_{l}}{({B}_{l}^{0}-{B}_{l})}^{2}$$

The above equation (3) has been mathematically fit to the BFR data (${B}_{l}^{0}$) reported by Srinivasan *et al*.^7^ for a tibial section (1.8 mm proximal to tibia-fibula junction) of a 16-week old female C57BL/6 mice as a response to a cantilever loading. The mathematical software SageMath Ver. 6.6^27^, in particular the “find_fit” function and Levenberg-Marquardt algorithm^28,29^, was used to fit the data by minimizing mean square error between the mathematical model and the experimental data. Equation (2) will be referred to as “Average BFR Model”, results for which is given in Section 3.1.1.

Note that the parameter *p* will be different for endocortical and periosteal surfaces, as endocortical surfaces have relatively lower strain. *p* will also be different for different loading conditions (e.g. axial loading^6,30,31^, cantilever loading^7,32^, three-point bending^21,33^, four-point bending^22,34^), as different loading cases have different strain distribution. A refined model is, therefore, needed which is valid for any loading case. Accordingly a “Site-Specific Model” has been developed, which will use the other four parameters identified here, viz. *ε*_*thres*_, *η*, *q* and *β*, as described in the next section.

### The Site-Specific Model

The stimulus at an individual osteocyte is computed similar to that in equation (1) except that *ε*_peak_ and Δε_max_ now correspond to the local strains experienced by the osteocyte in consideration. Accordingly, stimulus at *i*-th cell is proposed to be

4 $${s}_{i}=h({\varepsilon }_{peak}^{i}-{\varepsilon }_{thres})\xi (\eta ){N}^{q}{d}^{\beta }\,{\rm{for}}\,{\varepsilon }_{peak}^{i}\ge {\varepsilon }_{thres}\,{\rm{and}}\,{\rm{if}}\,{\rm{the}}\,i-\mathrm{th}\,{\rm{cell}}\,{\rm{is}}\,{\rm{an}}\,{\rm{osteocyte}},=0,{\rm{otherwise}}\,{\rm{or}}\,{\rm{for}}\,{\rm{an}}\,\mathrm{osteoblast},$$

where ${\varepsilon }_{peak}^{i}=max(|\varepsilon ({x}_{i},{y}_{i},t)|)$ is the peak strain experienced by the *i*-th cell. (*x*_*i*_, *y*_*i*_) is the coordinates of the *i*-th cell. *h* is a constant to be determined. Parameters *ε*_*thres*_, *η*, *q* and *β* are already determined by the Average BFR Model (Section 2.1).

The stimuli are proposed to diffuse through the osteocyte-osteoblast network (Fig. 1(E)). The stimuli finally reach the osteoblasts, which in turn produce new bone proportional to the stimuli reaching them.

Assuming Ca^2+^ to be the secondary stimulus as a response to mechanical loading^7^, *h* in equation (4) relates to the increase in Ca^2+^ concentration in cytoplasm of osteocytes as a response to mechanical loading. Accordingly, *s*_*i*_ presents the ‘strength’ of calcium signaling, which is a function of strain and its number of cycles. The value of *h* is not critical for this study as the value is not explicitly needed. One way to define *h*, however, would be the peak amplitude of Ca^2+^ concentration spike with respect to the baseline, when bone matrix around the osteocyte is loaded with a single square pulse of loading of 1s duration producing 1 με more than the threshold strain for bone formation. Then *h* can be roughly estimated by studying Ca^2+^ response to a single cycle of loading such as in Jing *et al*.^16^, where applying 1546 με to the bone matrix around an osteocyte results in rougly trianglular spike of Ca2+ concentration in cytosol with respect to its baseline. This spike has approximate amplitude of 1.7 times the baseline concentration. Assuming this baseline to be 50 nM (as for rat osteoblasts reported by Donahue *et al*.^17^), the spike amplitude would be 85 nM. *ξ*(*r*) = 1 approximately for this case because of rest-inserted loading, similar to what is expected in case of a single pulse loading. *h* is thus simply the ratio of the peak concentration and strain excess to the threshold (i.e., 1546–856 με). The value of *h* would, therefore, be 0.123 nM/με.

The stimuli produced inside the cells are assumed to ‘diffuse’ through each of the one-dimensional osteocytic processes and follow the Fick’s first law for steady-state diffusion:

5 $$J=-\,D\frac{d\psi }{dx}$$

where *J* is the diffusion flux (mols of Ca2+ diffusing per unit area per unit time), *D* is the diffusivity (a constant, which may be assumed to be 5.3 × 10^−6^ cm^2^ s^−1^ as measured by Donahue and Abercrombie^18^ for Ca2+ *in vivo*), *ψ* is the concentration of the stimulus (mols per unit volume) and *x* is the position along the cell process. This differential equation may be solved using a finite element method and may be accordingly discretized as follows:

6 $${\bf{Q}}={\bf{K}}\,{\boldsymbol{\Psi }}$$

where **Q** is a vector containing stimulus flow rate (mols of Ca^2+^ per unit time) at nodes and **Ψ** is vector containing the nodal concentration of the stimuli. **Ψ** includes the stimulus concentration computed earlier (*s*_*i*_) using equation (4), i.e. *ψ*_*i*_ = *s*_*i*_. **K** is the diffusion stiffness matrix assembled from stiffness matrix of individual osteocytic processes ***k***_*j*_. ‘Stiffness’ of the *j*th process is taken as

7 $${{\boldsymbol{k}}}_{j}=\frac{D{A}_{j}}{{L}_{j}}[\begin{array}{cc}1 & -1\\ -1 & 1\end{array}]$$

where *A*_*j*_ and *L*_*j*_ are cross-sectional area and length of *j*th process. It has been assumed that every osteocytic cell process has a uniform cross-sectional area of *A*^0^ = 0.025 μm^2^ ^19^.

The stimulus should be fully ‘utilized’ by the osteoblasts to form new bone. The nodal concentrations of the stimulus (*ψ*_*i*_) at the osteoblasts are thus assumed to be zero. The corresponding stimulus flow rate (*q*_*i*_) at the osteoblasts will be negative as osteoblasts act like a sink for the osteogenic stimuli.

The mineral apposition rate (MAR) at an osteoblast is assumed to be proportional to the stimulus flow rate (*q*_*i*_) at the osteoblast in consideration. In other words, MAR of *i*-th osteoblast is given by

8 $${m}_{i}=k\,{q}_{i}$$

where *k* is the constant of proportionality to be determined by fitting this mathematical model to experimental data. *k* is a negative real number as *q*_*i*_ at osteoblasts is also negative. Due to linearity of equation (8) and proportionality of *s*_*i*_ to *h*, the stimulus flow rate *q*_*i*_ will vary directly proportional to *h*, if other parameters are kept the same. Similarly, *q*_*i*_ is also directly proportional to *D*. The values of *h* and *D* are therefore not critical, as the process of curve fitting will automatically find a suitable *k* to get the same *m*_*i*_. The value of *k* is that minimizes the following error-squared function *φ*(*k*):

9 $${\rm{\phi }}(k)={\sum }_{i=1}^{{n}_{b}}{({m}_{i}^{0}-k{q}_{i})}^{2}$$

where ${m}_{i}^{0}$ is the experimental mineral apposition rate (MAR) at *i*-th osteoblast and *n*_*b*_ is the total number of osteoblasts. The value of *k* is obtained by linear regression:

10 $$k=\frac{{\sum }_{i=1}^{{n}_{b}}{m}_{i}^{0}.{q}_{i}}{{\sum }_{i=1}^{{n}_{b}}{q}_{i}^{2}}$$

If ${m}_{i}^{0}$ is not known and instead the overall (i.e. average) bone formation rate (*B*) for the section is given to be *B*^0^, then *k* can be computed by using the following relationship:

11 $$B=\frac{k{\sum }_{i=1}^{{n}_{b}}{q}_{i}}{{n}_{b}}$$

If *n*_*l*_ number of loading protocols are used as mentioned in Section 2.2, the optimal value of *k* is obtained by minimizing the following squared error:

12 $${\rm{\Phi }}(k)={\sum }_{l=1}^{{n}_{l}}{({B}_{l}^{0}-{B}_{l})}^{2}={\sum }_{l=1}^{{n}_{l}}{({B}_{l}^{0}-\frac{k{\sum }_{i=1}^{{n}_{b}}{q}_{i}^{l}}{{n}_{b}})}^{2}$$

where ${q}_{i}^{l}$ is the stimulus flow rate at *i*-th osteoblast for *l*-th loading protocol. *k* is accordingly obtained as

13 $$k=\frac{{n}_{b}{\sum }_{l=1}^{{n}_{l}}({B}_{l}^{0}{\sum }_{i=1}^{{n}_{b}}{q}_{i}^{l})}{{\sum }_{l=1}^{{n}_{l}}{({\sum }_{i=1}^{{n}_{b}}{q}_{i}^{l})}^{2}}$$

**Figure 2:**
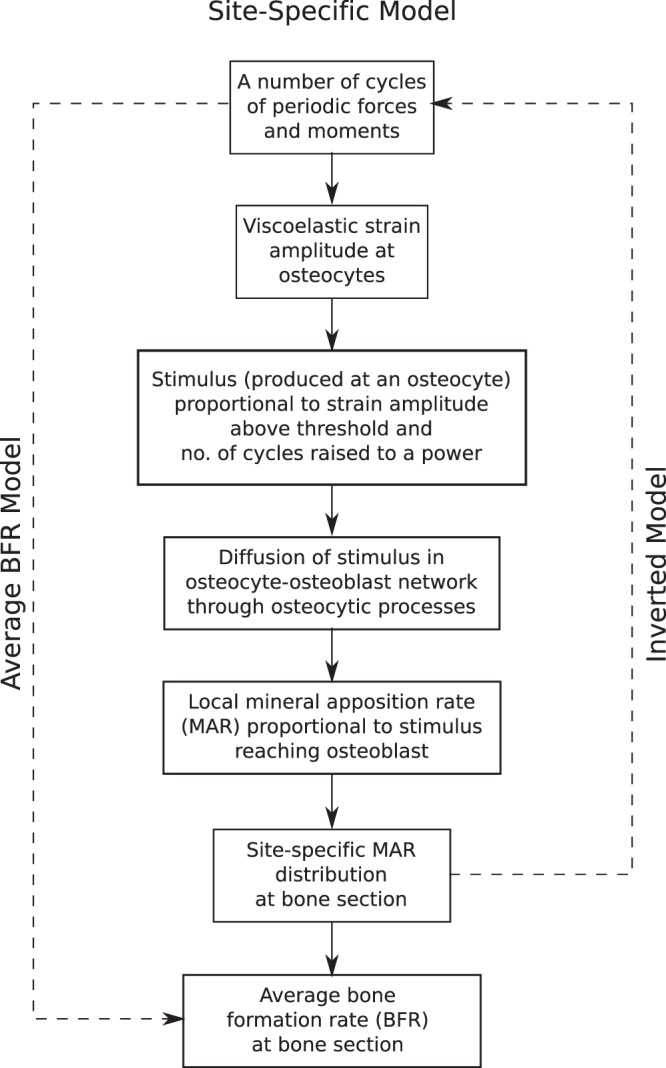
Flowchart of the site-specific model and its relation to the average BFR model and the inverted model. Given the number of cycles of a loading waveform, the site-specific model computes site-specific MAR distribution as well as average BFR at periosteal surface. The inverted model reverses the problem, i.e. it finds the loading parameters needed to achieve a prescribed site-specific MAR distribution. The average BFR model computes average BFR/BS at the section of interest based on loading parameters (including resulting peak viscoelastic strain) without computing osteocyte-level stimulus and its diffusion in osteocyte-osteoblast network.

In absence of sufficient site-specific new bone formation data, the constant *k* has been determined using overall BFR data (${B}_{l}^{0}$) given in Srinivasan *et al*.^7^ for 16-week old wild-type (C57BL/6) female mice. This site-specific model has been summarized in the flow chart given in Fig. 2. The results for this model are given in Sections 3.1.2 to 3.1.4 for different loading conditions.

### The Inverted Model

The current model is invertible, i.e. if site-specific MAR is prescribed, it is possible to easily find the normal force and bending moment (or alternatively strain distribution) at the section that would give the prescribed site-specific new bone formation (Fig. 2). If ${m}_{j}^{0}$, *j* = 1 to ${n}_{p}\le {n}_{b}$, is the desired mineral apposition rate at *j*-th chosen osteoblast, *m*_*j*_ is the corresponding computationally obtained MAR; and *BFR*^0^ and *BFR* are the desired and computational bone formation rates, respectively, then the following error squared function is to be minimized by varying amplitudes of normal force (${F}_{z}^{0}$), bending moment about medial-lateral axis (${M}_{x}^{0}$) and bending moment about anterior-posterior axis (${M}_{y}^{0}$) for a prescribed waveform of unit amplitude (*u*(*t*)) and number of cycles (*N*) of this waveform:

14 $${\rm{g}}({F}_{z},{M}_{x},{M}_{y})=\lambda {({B}^{0}-B)}^{2}+\sum _{j=1}^{{n}_{p}}{({m}_{j}^{0}-{m}_{j})}^{2}$$

where *m*_*j*_ = *kq*_*j*_ is obtained by solving equation (6), i.e. **Q** = **K ψ**. Stimulus concentration matrix **Ψ**, stiffness matrix **K** and stimulus flow rate **Q**, are computed as described earlier. **Ψ** is composed of *s*_*i*_ computed as per equation (4), where *h*, *ε*_*thres*_, *η*, *q* and *β* are known constants from the Site-Specific Model (Section 2.2). Number of cycles, *N*, is to be prescribed. *B* is computed using equation (11). λ is a Lagrange multiplier, which can be chosen based on relative weightage of the overall BFR equality constraint, i.e. *B* = *B*^0^. If *B*^0^ is not known, then *λ* = 0. As there are three unknowns, at least three of *B*^0^ and ${m}_{j}^{0}$’s should be known/prescribed for a unique solution. Since the unit waveform (*u*(*t*)) is prescribed, *F*_*z*_, *M*_*x*_, and *M*_*y*_ are given by:

15 $${F}_{z}(t)={F}_{z}^{0}u(t)$$

16 $${M}_{x}(t)={M}_{x}^{0}u(t)$$

17 $${M}_{y}(t)={M}_{y}^{0}u(t)$$

The objective function g(*F*_*z*_, *M*_*x*_, *M*_*y*_) may be minimized by using a standard optimization method with a reasonable initial guess for ${F}_{z}^{0}$, ${M}_{x}^{0}$ and ${M}_{y}^{0}$, whose optimal values are obtained after a number of iterations. We used the mathematical software SciLab^35^, in particular the “lsqrsolve” function to get the optimal values for ${F}_{z}^{0}$, ${M}_{x}^{0}$ and ${M}_{y}^{0}$ using Levenberg-Marquardt algorithm^28,29^. One example has been solved using this inverted model and the corresponding results are shown in Section 3.1.4.

### Statistical Tests

The new bone formation predicted by the developed mathematical model has been evaluated with respect to experimental findings by two statistical tests, viz. Student’s t-test and Watson’s U^2^ test^36,37^. A one-sample two-tailed t-test has been used to compare the average bone formation rate per unit bone surface (BFR/BS) values, while a circular goodness-of-fit test viz. Watson’s U^2^ test has been used to compare site-specific MAR. Watson’s U^2^ test is used to compare two circular probability distribution functions^36,37^. Probability distribution function (PDF) for the experimental results is assumed to be proportional to experimental MAR distribution. Similarly, PDF for the mathematical model predictions is assumed to be proportional to the predicted MAR distribution. Both t-test and Watson’s U^2^ test have been done using Scilab^35^ programming. The Watson’s test has been done based on works by Watson^36^ and Stephens^38^.

## Results

### Lamellar Bone Adaptation at Periosteal Surface

#### The Average BFR Model: Cantilever Loading

**Figure 3:**
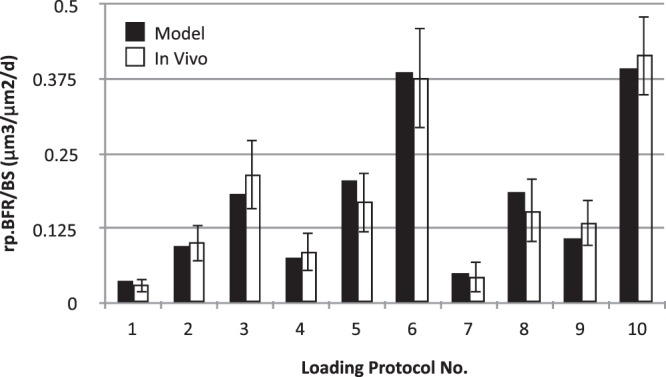
Comparison between the mathematical model and the *in vivo* experiment for new bone formation. Relative periosteal bone formation rate (rp.BFR/BS) obtained by the mathematical model (black bars) is statistically equal to that obtained by *in vivo* experiments for each of the 10 protocols used by Srinivasan *et al*.^7^.

The developed theory has been tested for cantilever bending^7,32^. In particular, periosteal *in vivo* data for tibia of 16 week old female C57BL/6 mice for the ten loading protocols obtained from Srinivasan *et al*.^7^ have been used to calibrate the developed model. The loading waveform used in the original *in vivo* study was trapezoidal, starting from 0 to the peak in 0.1 s, holding at the peak for 0.8 s and decreasing linearly to 0 in 0.1 s. In some of the protocols, there was 10 s rest given between two loading waveforms. The peak strain varied (viz. 1000 με, 1250 με, 1600 με) among the protocols and so did the number of cycles (viz. 10, 50, 250). The loading was given three alternate days in a week, for 3 weeks. All mice received calcein labels on days 10 and 19 (day 1 corresponded to first loading). Values of mid-diaphysis section properties – *A*, *I*_*x*_, *I*_*y*_ and *I*_*xy*_ have also been taken from Srinivansan *et al*.^7^. After curve fitting according to equations^27–29^, the parameters that fit the BFR data in Srinivasan *et al*.^7^ at mid-diaphyseal cross-section 1.8 mm proximal to tibia-fibula junction are as follows: *p.d*^*β*^ = 1.06859 × 10^−4^ μm/day/με, *q* = 0.404736, *r* = 2π*η*/*E* = 0.436235 s, and ε_*thres*_ = 856.126 με. As *d* = 3 for all protocols, *β* could not be separately determined and hence the value of *p.d*^*β*^ has been computed. As shown in Fig. 3, the model BFR/BS values are found to be not significantly different from the mean experimental values (p > 0.48 for every protocol).

#### Site-Specific Model: Cantilever Loading

**Figure 4:**
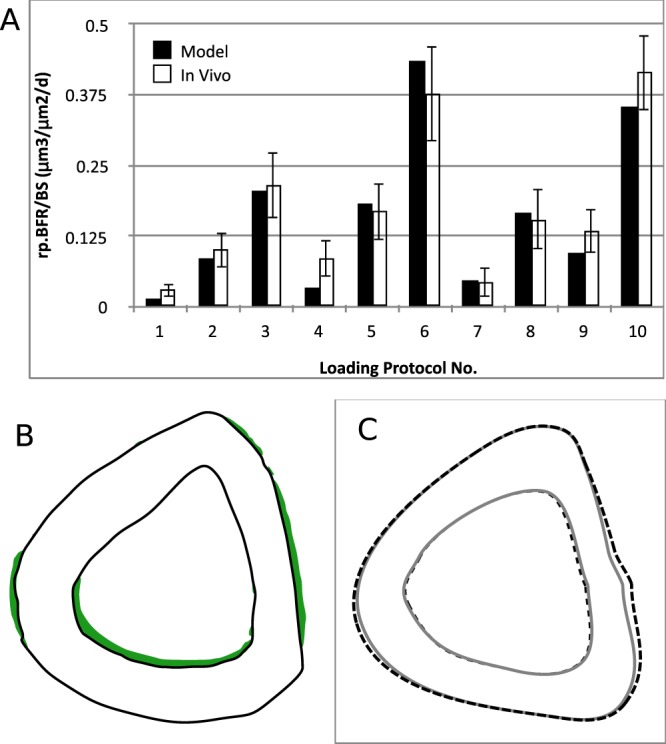
Comparison of new bone formation between the site-specific model and the *in vivo* experiment. (**A**) Periosteal bone formation rate obtained by the site-specific mathematical model (black bars) is not significantly different from that of *in vivo* experiments for each of the 10 protocols used by Srinivasan *et al*.^7^. (**B**) *In vivo* distribution of new bone formation for Protocol 6 is shown in green, which is adapted from Srinivasan *et al*.^7^. (**C**) Outer boundaries of periosteal and endocortical osteogenesis predicted by the mathematical model for Protocol 6 are shown as dashed line.

Using the same *in vivo* data used in Section 3.1.1, the site-specific model has been established by finding *k* according to equation (13). The locations of osteocytes and osteoblasts on the tibial cross-section and their network have been taken from the same source. The value of the product of *k*, *h*, *D*, *A*^0^ and *d*^*β*^ has been approximately found to be *khDA*^0^*d*^*β*^ = −1.8563 × 10^−3^ μm^2^ day^−1^με^−1^ (where *d* = 3) i.e. *khDA*^0^ = −1.8563 × 10^−3^/3^β^ μm^2^ day^−1^με^−1^. As compared in Fig. 4(A), the BFR/BS predicted by the site specific model is not significantly different from the *in vivo* data (p > 0.15 for every protocol; average *p*-value = 0.576 for the ten protocols).

The *in-vivo* new bone formation is approximately as shown in Fig. 4(B) for loading protocol no. 6 of Srinivasan *et al*.^7^. This loading protocol has peak strain (ε_*peak*_) of 1600 με, rest of 10 s and number of cycles (*N*) of 50. The corresponding periosteal osteogenesis predicted by the site-specific model for the same loading protocol is shown in Fig. 4(C). The total BFR/BS is 0.436 μm^3^/μm^2^/day, which is close to the experimental value of 0.377 ± 0.084 (SE) μm^3^/μm^2^/day (*p* = 0.51, t-test). The predicted MAR distribution is also significantly close to that of the experimentally obtained new bone distribution (*p* = 0.99 for Watson’s U^2^ test^36^).

#### Site-Specific Model: Axial Loading

**Figure 5:**
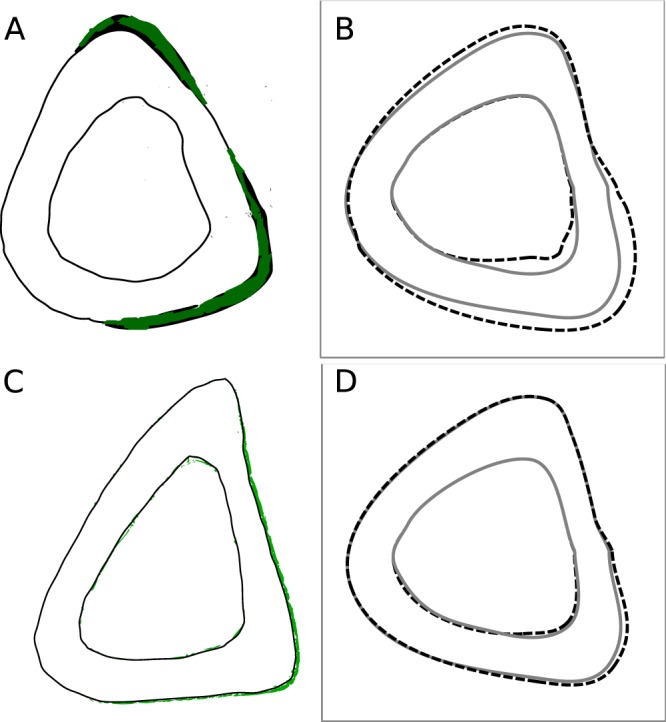
Comparison of site-specific new bone formation between experimental and mathematical models in axial loading cases. *In vivo* bone section showing new formation in green which has been adapted from (**A**) Weatherholt *et al*.^31^ and (**C**) Mahaffey *et al*.^39^. Dashed lines in (**B**) and (**D**) respectively show the corresponding new bone formation predicted by the mathematical model.

**Figure 6:**
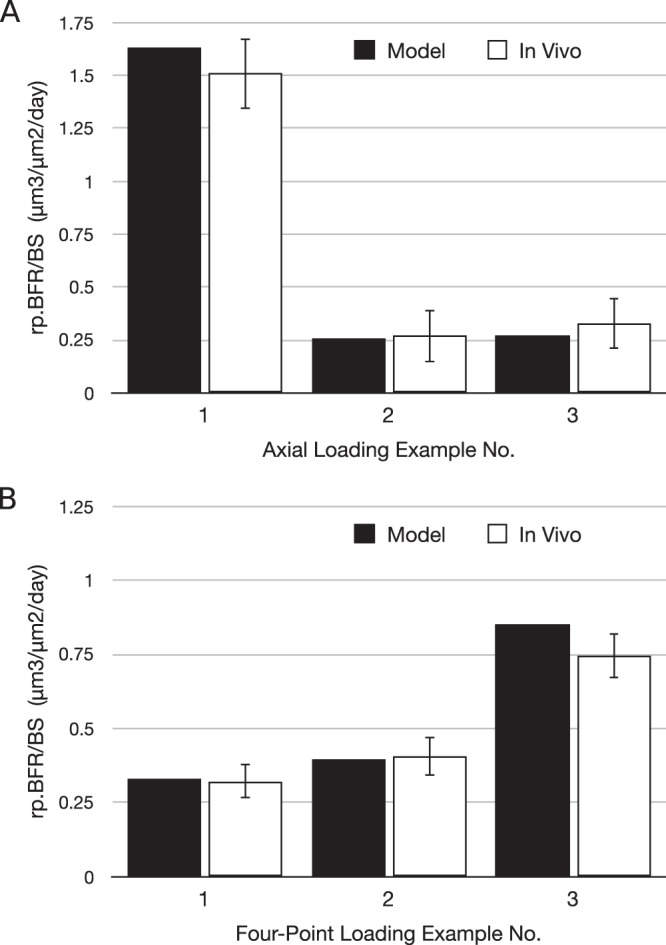
Comparison of relative periosteal bone formation rates (rp.BFR/BS) between model predictions and actual experimental values. (**A**) Axial loading examples 1, 2 and 3 have been taken from Weatherholt *et al*.^31^, Mahaffey *et al*.^39^ and Willie *et al*.^43^, respectively. (**B**) Four-point bending examples have been taken from Turner *et al*.^22^ (examples 1 and 2) and Kuruvilla *et al*.^40^ (example 3).

**Example 1:** The first axial loading example solved is that from Weatherholt *et al*.^31^, where tibia of 16 week old female C57BL/6 mice underwent axial loading of 7N magnitude having 360 cycles of 2 Hz haversine waveform for 4 weeks at the rate of 3 days a week. Strain distribution is kept similar to Weatherholt *et al*.^31^ (e.g. 1833 με at medial side). The model parameters i.e. *k*, *h*, *D*, *A*^0^ and *d*^*β*^ have been kept the same as that in the cantilever loading example (Section 3.1.2). The experimental and predicted new bone formations are shown in Fig. 5(A,B), respectively. The predicted BFR/BS is 1.63 μm^3^/μm^3^/day, which is not significantly different from the experimental value of about 1.5 ± 0.17 (SE) μm^3^/μm^3^/day (Fig. 6(A), *p* = 0.456). The predicted site-specific MAR distribution (Fig. 5(B)) on the periosteal surface is close to that in the experimental case (Fig. 5(A)) (p = 0.997).

**Example 2:** The second axial loading example solved is from Mahaffey *et al*.^39^, where 16 week old female C57BL/6 mice were loaded with 60 cycles of 2 Hz haversine waveform of 5N magnitude for 2 weeks at the rate of 3 days per week. The strain distribution is kept similar to that of the experimental study^39^, e.g. the strain is about 1200 με at anteromedial aspect of mid-diaphysis. The new bone formation experimentally obtained and that predicted by the model are shown in Fig. 5(C,D), respectively. The BFR/BS predicted by the model is 0.26 μm^3^/μm^3^/day, which is close to the experimental value of 0.27 ± 0.12 μm^3^/μm^3^/day (Fig. 6(A)) (p = 0.936). The predicted MAR distribution (Fig. 5(D)) is not significantly different from the *in vivo* study (Fig. 5(C)) (p = 0.994 for Watson’s U^2^ test). Some difference in MAR distribution may be attributed to the difference in shape of the cross-section.

**Example 3:** Another axial loading example is taken from the work of Willie *et al*.^30^, where tibiae of 26 week old female C57Bl/6 J mice were loaded with 216 cycles of 4 Hz triangular waveform with 11N peak compressive load for 2 weeks at the rate of 5 days per week. The waveform had a 5 s rest period inserted after every 4 cycles. The strain distribution is similar to that in the original experimental study^30^, e.g. the peak tensile and compressive strains are approximately 1080 and −1695 με, respectively. The model parameters *k*, *h*, *D*, *A*^0^ are the same as that in Examples 1 and 2 in this section, whereas *β* = 0.465 has been used in accordance with Section 3.1.4. The predicted BFR/BS (0.27 μm^3^/μm^3^/day) is close to that experimentally obtained value of 0.33 ± 0.12 (SE) (Fig. 6(A)) (p = 0.63 for t-test).

#### Site-Specific Model: Four-Point Loading

**Examples 1 and 2:** The site-specificity of the model was further tested for the four-point bending case reported by Turner *et al*.^22^. The original study was for tibia of 9**-**month-old female Sprague-Dawley rats. The loading waveform was sinusoidal with 2 Hz frequency, applied for 36 cycles a day for 14 days. The rats received calcein label on days 5 and 12 (day 1 corresponds to the first day of loading). The study lasted for 14 days. The force amplitudes were 27 (Example 1) and 33N (Example 2), which resulted into peak strains of 1404 and 1484 με, respectively. Applying these loading protocol including the strain distribution, the mathematical model was fitted to experimental BFR. The corresponding *khDA*^0^*d*^*β*^ value is found to be −2.7527 × 10^−3^ (where *d* = 7) i.e. *khDA*^0^ = −2.7527 × 10^−3^/7^β^ μm^2^ day^−1^με^−1^. Equating *khDA*^0^ for the cantilever and four-point bending cases, we get *β* = 0.465, which is slightly more than the value of *q* (=0.405) given in Section 3.1.1. Based on the estimated values of *h*, *D*, and *A*^0^ given earlier, the value of *k* would accordingly be 7.908 × 10^6^ μm/nmol. The corresponding BFRs computed by the model are 0.330 and 0.396 μm^3^/μm^2^/day, respectively, which are close to the experimental values 0.321 ± 0.058 (SE) and 0.408 ± 0.065 μm^3^/μm^2^/day (Fig. 6(B)) (p = 0.875 and 0.858, respectively). Unfortunately, no histological section showing lamellar new bone formation or site-specific MAR data are available for these loading cases, so the predicted site-specific MAR could not be tested against the experimental values.

**Example 3:** This 4-point bending example is taken from the work of Kuruvilla *et al*.^40^, where tibiae of 16 week old C57BL/6 J mice were loaded with 99 cycles of 2 Hz haversine waveform per day for 3 weeks at the rate of 3 days a week. The strain distribution is also kept similar to that in the experimental study^40^, e.g. approximately 2000 με at the lateral surface of tibia. The predicted BFR/BS is 0.853 μm^3^/μm^2^/day, while the experimental value is 0.745 ± 0.076 μm^3^/μm^2^/day (Fig. 6(B)) (p = 0.195). The predicted new bone formation primarily at the lateral surface of the section is in accordance with the incomplete histological section shown by Kuruvilla *et al*.^40^.

#### The Inverted Model: Axial Loading

The average BFR is invertible as it is an explicit algebraic function (equation (1)). The peak strain required to achieve a desirable overall BFR may be given by:

18 $${\varepsilon }_{peak}={\varepsilon }_{thres}+\frac{B}{p\xi (\eta ){N}^{q}{d}^{\beta }}$$

**Figure 7:**
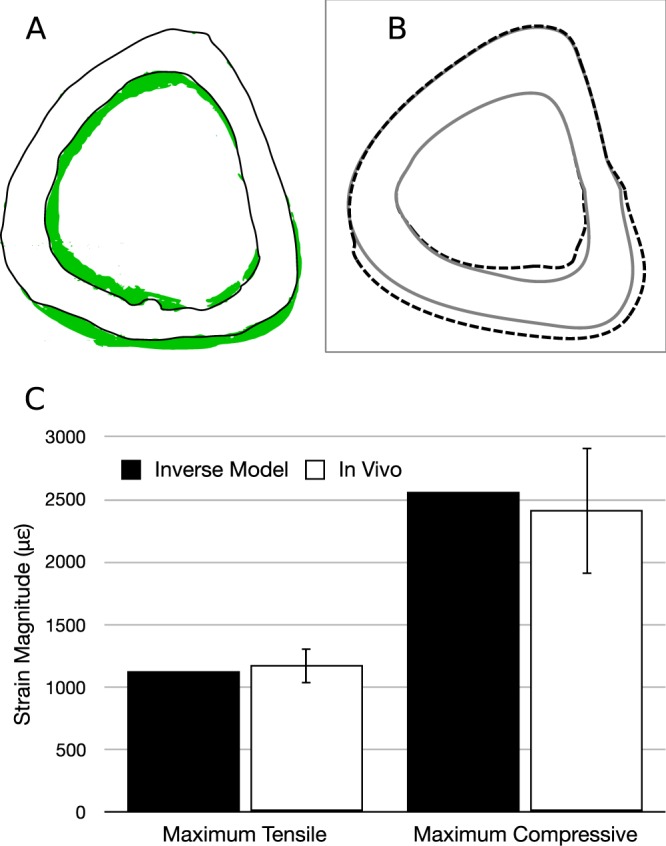
*In vivo* osteogenesis due to axial loading versus that predicted by the inverse model. (**A**) For axial loading condition^6,41–43^, *in vivo* new bone formation is roughly shown in green, which is adapted from Willie *et al*.^43^. (**B**) The computational new bone formation corresponding to the forces predicted by the ‘inverse’ mathematical model is shown by dashed lines. (**C**) The peak magnitude of tensile and compressive strains predicted by the mathematical model have been compared to the respective experimental values.

The site-specific osteogenesis can also be easily inverted using optimization methods^28,29,35^. As the axial loading on mouse tibia has been extensively studied earlier^6,14,30,31,41–44^, the inverted mathematical model has been tested for the axial loading case, viz. with respect to the *in vivo* studies by Willie *et al*.^30^. The corresponding experimental new bone formation shown in Fig. 7(A) is for 10-week-old female C57BL/6 mice. The loading waveform used for this study was the same as that given in Section 3.1.3 (Example 3).

We prescribed periosteal MAR values approximately similar to that (relative to experimental control) in Willie *et al*.^30^, as roughly shown in Fig. 7(A). In particular, one osteoblast at the posterior-medial vertex, one at posterior side and one at posterior-lateral vertex of the bone cross-section are prescribed MAR of 0.9, 2.7 and 0.9 μm/day, respectively. Additionally, one osteoblast each at the lateral side, anterior vertex, and medial side is also prescribed MAR of 0 μm/day. The waveform is kept approximately the same as that for corresponding *in vivo* study, except that the amplitude of the waveform is unity. The number of cycles *N* is also kept the same (216 per bout). After optimization, we get the sectional forces (${F}_{z}^{0}$, ${M}_{y}^{0}$ and ${M}_{x}^{0}$) that produce peak tensile and compressive strains of 1127 and −2563 με, respectively, which are close to 1174 ± 140 (SE) and −2410 ± 500 με (Fig. 7(C)) (p = 0.743 and 0.766, t-test), respectively resulting from ${F}_{z}^{0}=11N$ of axial loading. The site-specific bone formation due to the predicted loading is shown in Fig. 7(B), which is site-specifically, approximately similar to that of the *in vivo* case (*p* > 0.99 for Watson’s U^2^ test). The BFR/BS computed by the model (0.884 μm^3^/μm^2^/day) is also close to the experimental value 0.930 ± 0.145 μm^3^/μm^2^/day (*p* = 0.760). The predicted neutral axis is approximately horizontal (${\tan }^{-1}({M}_{y}^{0}/{M}_{x}^{0})=3^\circ$) as also evident in the original *in vivo* study^30^ and other works in the literature involving axial loading of mouse tibia^6,45^.

### Lamellar Bone Adaptation at Endocortical Surface

#### Cantilever Loading

Although the endocortical bone formation data are not studied in Srinivasan *et al*.^7^, the comparison has been approximately made to only one section shown there. Based on the model parameters given in Section 3.1.2, the predicted new bone distribution at the endocortical surface is also shown in Fig. 4(C), which is not significantly similar to the experimental distribution (p = 0.89 for Watson’s U^2^ test). The predicted BFR/BS is 0.132 μm^3^/μm^2^/day, which is less than estimated experimental BFR/BS of 0.5 μm^3^/μm^2^/day. This is in accordance with the literature that endocortical surface is more mechano-responsive than the periosteal surface due to extra recruitment of osteoblasts from bone marrow^10,39,44,46^. As such, endocortical surfaces remodel differently^10,47^ e.g. endocortical remodeling depends on age of animal more than periosteal remodeling does^44^. The periosteal new bone formation is, therefore, more consistent than endocortical new bone formation, as also evident from other works of Srinivasan *et al*.^48,49^. The current model does not take into account of such differences between the two surfaces.

#### Four-Point Loading

In continuation to and for the model parameters given in Section 3.1.4, the endocortical BFR/BS computed by the model are 0.080 μm^3^/μm^2^/day (*p* = 0.92) and 0.112 μm^3^/μm^2^/day (*p* = 0.01), respectively. The corresponding experimental values are 0.085 ± 0.052 (SE) and 0.041 ± 0.019 μm^3^/μm^2^/day, respectively^22^. This discrepancy between model prediction and experimental values may be due to the bone resorption at endocortical surfaces in adult rodents^44^, similar to that observed in human adults as well^50^. The current model does not take into account for the natural bone resorption at the endocortical surfaces.

### Woven Bone Adaptation

#### Four-Point Loading

**Figure 8:**
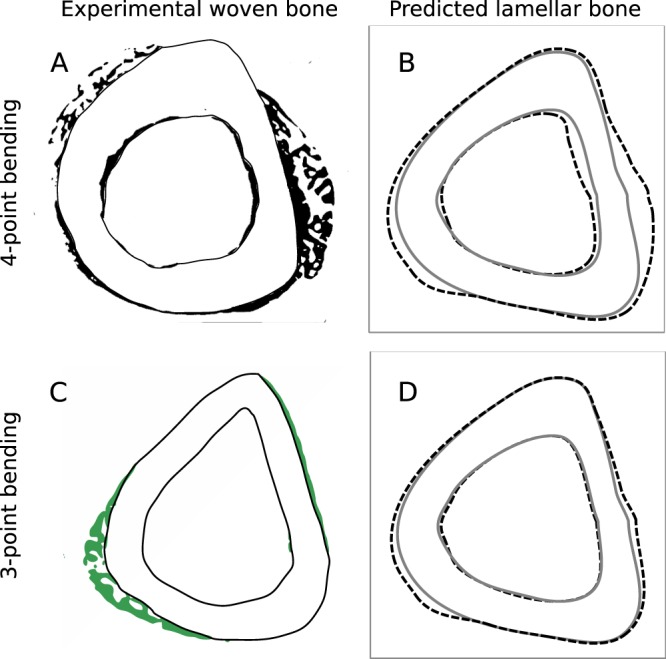
*In vivo* new bone formation for 4-point and 3-point bending versus that predicted by the model. (**A**) The *in vivo* new bone formation for 4-point loading condition is shown in solid black, which is adapted from Turner *et al*.^22^. (**B**) For 4-point bending^22,34^, periosteal and endocortical new bone obtained from the model is shown with dashed lines. (**C**) The *in vivo* new bone formation for 3-point loading is shown in green, which is adapted from Sakai *et al*.^21^. (**D**) For 3-point bending condition^21,33^, periosteal and endocortical new bone formation predicted by the model is shown by dashed lines.

Turner *et al*.^22^ show a section corresponding to 64N load which induces 3010 με peak strain and results in woven bone formation at periosteal surface (Fig. 8(A)). Note that the current model does not account for woven bone formation, which is regulated by a different osteogenic mechanism and results in elevated bone formation^22,42,51^. Hence, based on the model parameters given in Section 3.1.4 (Examples 1 and 2), the predicted periosteal new bone formation (1.768 μm^3^/μm^2^/day) is very less than the estimated experimental elevated BFR/BS of 8.563 μm^3^/μm^2^/day. The model’s site-specificity prediction is shown in Fig. 8(B). Both (the *in vivo* experiment and the model) have new bone formation at the lateral and medial sides. The *in vivo* case additionally has woven bone formed as anterior-medial side, which is also present in the sham loading case. The new bone distribution relative to the sham loading was found to be close to that predicted by the present model (*p* > 0.99, Watson’s U^2^ test).

The endocortical new bone formation rate (BFR/BS) for 65N load is predicted to be 0.916 μm^3^/μm^2^/day, which is significantly less than the experimental value of 2.466 ± 0.173 μm^3^/μm^2^/day (p < 0.001) due to woven bone formation. The predicted MAR distribution is similar to that of experimental distribution (p > 0.99).

#### Three-Point Loading

The original *in vivo* study reported by Sakai *et al*.^21^ is for 3-point loading of tibia of 10-week-old male C57BL/6 mice. The loading waveform was rectangular pulse of 6N amplitude and 0.5 s duration followed by 39.5 s rest. The tibia was loaded for 36 cycles each day for three alternate days in the first week only. The peak strain was 2100 με. There was no loading in the second week. The mice received Calcein labels on days −2, 3, 8, and 13 (day 0 being the first loading day). The study was conducted only for 2 weeks. The corresponding site-specific new bone formation is shown in Fig. 8(C). Note that the shown section has woven bone formation, which has not been incorporated in the current model. Keeping the same peak strain, orientation of the neutral axis and loading waveform as that of the *in vivo* study, the developed model predicts site-specificity as shown in Fig. 8(D), which is approximately similar to that of the *in vivo* bone formation (p > 0.99, for both periosteal and endocortical surfaces for Watson’s U^2^ test). Both have new bone formation on medial and lateral sides.

### Summary of Results and Future Work

- The newly developed mathematical model is able to predict lamellar periosteal BFR/BS and site-specific MAR for not only cantilever loading but also for axial loading cases, as evident from Section 3.1.1 to 3.1.3.
- For four-point loading case (Section 3.1.4), the model also works for predicting lamellar periosteal BFR/BS, while site-specificity could not be tested due to unavailability of data.
- As demonstrated in Section 3.1.5, the inverted model predicts the strain distribution that would produce the prescribed site-specific lamellar periosteal MAR and thus the force required for the desired new lamellar bone formation.
- As described in Section 3.2, the current mathematical model is unable to predict BFR/BS and site-specific MAR at endocortical surfaces. There are three reasons for that – (i) the endocortical surfaces respond differently than periosteal surfaces^10,44,47^, (ii) there is a continuous age-dependent bone resorption at endocortical surface in normal adults^44,50^, and (iii) endocortical surfaces are more responsive to exogenous loading than periosteal surface^39,44,46^. These have not been implemented in the current model and this has been acknowledged as a limitation.
- As discussed in Section 3.3, the model is also unable to predict woven BFR/BS and site-specific MAR at either of periosteal and endocortical surfaces. This is because the woven bone is formed by a different osteogenic mechanism than that of lamellar bone formation^22,42,51^.
- As such, new bone formation at endocortical surface is more complex than that at periosteal surface. Mathematical modeling of endocortical new bone formation itself would be more difficult and therefore has been taken as future work.
- Similarly, developing a model of woven bone adaptation itself would be a major task and hence would be taken as a future work.

## Discussion

The results establish that there are four main mechanical factors on which new bone formation depends. The first is how much the induced normal strain is above the threshold value (ε_thres_). This factor is responsible for why there is no bone formation when induced strains are within the threshold value. The second factor is the ratio of trough-to-crest amplitude of normal strain and the maximum magnitude of normal strain. This ratio brings out the difference between loading waveforms with and without rest inserted between two consecutive waveforms. Assuming bone to be simply elastic does not differentiate between these two kinds of loading waveforms, whereas assuming bone to be viscoelastic does. This emphasizes the role interstitial fluid flow plays in new bone formation. This accounts for why rest-inserted loading is more osteogenic than a loading without any rest. The third factor is the number of cycles of a waveform. Larger the number of loading cycles, the more is the new bone formation; however, the osteogenic potential of each loading cycle is less than that of the previous cycle. This is akin to the fatigue damage accumulation and hints at possible role of micro-cracks in new bone formation. The fourth factor is the number of days of loading per week. Larger the number of days, the greater is the new bone formation; however, osteogenic potential of each day of loading is less than the previous one. Factor *β* being slightly larger than the factor *q* indicates that daily loading is better than the same total number of cycles spread over less number of days.

The main feature of the developed model is that it is invertible, i.e. load magnitude and directions or alternatively, strain distribution on the cross-section can be found out that would induce a desired site-specific new bone formation. The ‘average BFR model’, ‘site-specific model’ and the ‘inverted model’ developed here may be extended for clinical purposes. For example, the magnitude and direction of mechanical loading may be in future, prescribed by orthopaedic surgeons for a desired overall or site-specific new bone formation. Researchers can also estimate loading for a desired BFR.

The average BFR model can be established for any kind of loading condition – cantilever, 3-point, 4-point or axial loading. For a given loading condition, there are five variables – peak strain, unit waveform, rest between two loading waveforms, number of cycles everyday and number of days of loading every week, out of which the peak strain can be found out applying equation (18). Alternatively, the number of cycles can be determined if the other four are prescribed along with the desired BFR.

For the given unit loading waveform, rest between two waveforms, the number of cycles everyday and the number of loading days per week, the inverted model is able to find the normal force (${F}_{z}^{0}$) and bending moments (${M}_{x}^{0}$,$\,{M}_{y}^{0}$) at the section that would get the desired new bone formation. This inverted model can be used when a given site-specific new bone distribution is desired. A small normal force will indicate no axial loading and the neutral axis in that case will pass approximately through the centroid of the section. The angle ${\tan }^{-1}({M}_{y}^{0}/{M}_{x}^{0})$ is the approximate orientation of the neutral axis to the medial-lateral direction. In order to get the given site-specific MAR, a bone is loaded such that the net normal force is ${F}_{z}^{0}$, bending moment is $\sqrt{{({M}_{x}^{0})}^{2}+{({M}_{y}^{0})}^{2}}$ and the neutral axis is approximately oriented ${\tan }^{-1}({M}_{y}^{0}/{M}_{x}^{0})$ angle to the medial-lateral direction.

In general, the developed mathematical model approximately predicted periosteal lamellar bone formation both in terms of the average BFR and site-specific MAR distribution. Some differences may be attributed to the difference in age, section shapes, calcein labeling days, and coordinates of osteocytes and osteoblasts. The model does not incorporate woven bone formation, age-related bone resorption and enhanced response of endocortical surface to exogenous loading, and, therefore, cannot predict woven bone formation rate and endocortical remodeling. The site specificity can, however, still be predicted for woven bone, as evident from the 3-point and 4-point loading cases described in Section 3.3.

## Conclusions

In summary, the elegant mathematical relationships developed here are robust as they approximately hold true for diverse loading conditions. These relationships are also invertible, which implies that it will make possible for the orthopaedic surgeons to readily compute and prescribe an exercise that would lead to a desired new bone formation. The current model can be improved further by incorporating natural endocortical resorption and woven bone formation at elevated strain environment. Even for lamellar bone formation, the model can be made more robust by taking into account the age of the mice, calcein labeling days, total weeks of loading, and also testing it for a large number of cases. For that, however, a very large number of experimental data will be needed covering variety of loading conditions, peak strains, loading waveforms, rest periods, number of cycles per loading bout, number of bouts per week, total number of loading weeks, period of calcein labeling, age of animals etc. In spite of unavailability of such large data in the literature, the current model attempts to connect presently available data for diverse loading conditions into a single mathematical model.

## Supplementary information

Supplementary Information

## Data Availability

All experimental data used in this work (such as the shape of bone cross-section, the locations of osteoblasts and osteocytes on the section, bone formation rate (BFR), site-specific MAR etc.) are from publicly available literature, as mentioned in the manuscript.
