# Supplementary material for: An Invertible Mathematical Model of Cortical Bone’s Adaptation to Mechanical Loading
Source: Sci Rep. 2019 Apr 10;9:5890. doi: 10.1038/s41598-019-42378-5 (PMC6458131; doi:10.1038/s41598-019-42378-5)
Supplement: Supplementary file 1 — Supplementary Information [file 41598_2019_42378_MOESM1_ESM.docx]

**Supplementary Information**

**Article in *Scientific Reports***

**An Invertible Mathematical Model of Cortical Bone’s Adaptation to Mechanical Loading**

Jitendra Prasad, and Ajay Goyal

**Supplementary Methods**

**S1. Osteogenic Stimulus**

Stimulus for new bone formation may be assumed to be analogous to cumulative fatigue damage ^1^. As per the fatigue failure theory ^2,3^, a material fails when the accumulated damage exceeds a threshold. For *N* number of cycles per day for *d* number of days of a fully-reversed sinusoidal loading $\sigma= \sigma_{a}sin\left( \omega t \right)$, the cumulative damage for the material with an endurance limit ($\sigma_{e}$) may is given by

$D=\left( \sigma_{a}-\sigma_{e} \right){(Nd)}^{q}=E\left( \varepsilon_{a}-\varepsilon_{e} \right)N^{q}d^{q}$ (1)

where *q* is a material constant and *E* is Young’s modulus. $\varepsilon_{a}$ and $\varepsilon_{e}$ are strains corresponding to stresses $\sigma_{a}$ and $\sigma_{e}$.

Analogous to the cumulative damage, the average relative periosteal bone formation rate (rp.BFR) has been proposed to be as follows:

$B=c\left( \varepsilon_{a}-\varepsilon_{e} \right)N^{q}d^{\beta}$ for $\varepsilon_{a}\geq\varepsilon_{e}$ , (2)

= 0, otherwise.

where *c*, $\varepsilon_{e}$ and *β* are constants to be determined. *β* may be equal to or a little different from the value of *q*.

To find the site-specific bone formation, the concentration of osteogenic stimulus produced at *i*-th cell is accordingly proposed to be:

$s_{i}=\alpha\left( \varepsilon_{a}^{i}-\varepsilon_{e} \right)N^{q}d^{\beta}$ for $\varepsilon_{a}^{i}\geq\varepsilon_{e}$ and if the *i*-th cell is an osteocyte,

$=0$, otherwise or for an osteoblast, (3)

where $\varepsilon_{a}^{i}$ is the amplitude of oscillatory strain. Assuming perfectly elastic material, the strain experienced by the *i*-th cell will be: $\varepsilon^{i}= \varepsilon_{a}^{i}sin\left( \omega t \right)$. *α* is a constant.

**S2. Viscoelastic Effects**

An in-vivo experiment for studying remodeling typically has a non-sinusoidal loading waveform acting *F*(*t*) acting on a long bone such as tibia and its osteogenic effect is measured at a cross-section of interest (Fig. 1). *F*(*t*) produces a force normal to the section *F_z_*(*t*); and bending moments about medial-lateral axis, *M_x_*(*t*) and about posterior-anterior axis, *M_y_*(*t*). According to the beam theory^3^, these normal force and bending moments produce normal stress as follows:

$\sigma(x,y,t)=\frac{F_{z}(t)}{A}-\left( \frac{I_{x}M_{y}(t)+I_{xy}M_{x}(t)}{I_{x}I_{y}-I_{xy}^{2}} \right)x+\left( \frac{I_{y}M_{x}(t)+I_{xy}M_{y}(t)}{I_{x}I_{y}-I_{xy}^{2}} \right)y$ (4)

where (*x*, *y*) are the coordinates of the point where normal stress is being calculated. *A* is the area of cross-section. *I_x_* and *I_y_* are second moment of area about x and y axes, respectively. *I_xy_* is the product of inertia for the cross-sectional area.

The stress may be expressed as a Fourier series as follows:

$\sigma(x,y,t)= \frac{a_{0}(x,y)}{2}+\sum_{n=1}^{\infty} \left[ a_{n}(x,y)\cos\left( \frac{2\pi nt}{T} \right)+b_{n}(x,y)\sin\left( \frac{2\pi nt}{T} \right) \right]$ (5)

where *T* is the time period of a complete cycle of the waveform *F*(*t*). The coefficients $a_{n}(x,y)$ and $b_{n}(x,y)$ are computed as follows:

$a_{n}(x,y)=\frac{2}{T}\int_{0}^{T} \sigma(x,y,t)\cos\left( \frac{2\pi nt}{T} \right)dt$ (6)

$b_{n}(x,y)=\frac{2}{T}\int_{0}^{T} \sigma(x,y,t)\sin\left( \frac{2\pi nt}{T} \right)dt$ (7)

To model the effect of interstitial fluid flow, the homogenized macroscopic behavior of bone is assumed to be a Kelvin-Voigt material. The resulting steady-state strain may be then given by

$\varepsilon(x,y,t)= \frac{a_{0}(x,y)}{2E}+\sum_{n=1}^{\infty} \frac{a_{n}(x,y)\cos\left( \omega_{n}t-\varphi_{n} \right)+b_{n}(x,y)\sin\left( \omega_{n}t-\varphi_{n} \right)}{\sqrt{E^{2}+{(\eta\omega_{n})}^{2}}}$ (8)

where *E* and *η* are modulus of elasticity and viscosity, respectively, and $\omega_{n}=2\pi n/T$. The phase lag ($\varphi_{n}$) is given by

$\varphi_{n}={tan}^{-1}\left( \frac{\eta\omega_{n}}{E} \right)$ (9)

If we define

$r=\frac{2\pi\eta}{E}$ (10)

Then

$\varepsilon(x,y,t)= \frac{a_{0}(x,y)}{2E}+\sum_{n=1}^{\infty} \frac{a_{n}(x,y)\cos\left( \omega_{n}t-\varphi_{n} \right)+b_{n}(x,y)\sin\left( \omega_{n}t-\varphi_{n} \right)}{E\sqrt{1+{(rn/T)}^{2}}}$ (11)

and

$\varphi_{n}={tan}^{-1}\left( \frac{rn}{T} \right)$ (12)

The peak-to-trough amplitude of the strain at point (*x*,*y*) may be given by

$\Delta\varepsilon(x,y)= max\left( \varepsilon(x,y,t) \right)-min\left( \varepsilon(x,y,t) \right)$ (13)

$\Delta\varepsilon(x,y)$ is thus a function of $r$, which is unknown to be determined. All other parameters required to calculate $\Delta\varepsilon(x,y)$ are known.

Further, we define the maximum peak-to-trough amplitude for the whole cross-section ($\Delta\varepsilon_{max}$) as follows:

$\Delta\varepsilon_{max}=max\left( \Delta\varepsilon(x,y) \right)$ (14)

In-vivo experiments typically report the peak strain ($\varepsilon_{peak}$) on the section, which may be defined as

$\varepsilon_{peak}= max\left( \left| \varepsilon(x,y,t) \right| \right)$ (15)

As the bone tissue is assumed to be a Kelvin–Voigt material, $\Delta\varepsilon_{max}$ and $\varepsilon_{peak}$ will occur at the same point (*x_e_* , *y_e_*), which corresponds to the furthest point on the cross-section from the neutral axis. The ratio of $\Delta\varepsilon_{max}$ to $\varepsilon_{peak}$ is represented as *ξ*(*r*) i.e.

$\xi\left( r \right)=\frac{\Delta\varepsilon_{max}}{\varepsilon_{peak}}$ (16)

To incorporate reduction of strain amplitude due to viscoelstic effects, we used $\xi\left( r \right)$ to approximately scale strain applitudes in equations (2) and (3). Equation (2) may be rewritten as:

$B=p\left( \varepsilon_{peak}-\varepsilon_{thres} \right)\xi\left( r \right)N^{q}d^{\beta}$ for $\varepsilon_{peak}\geq\varepsilon_{thres}$, (17)

= 0, otherwise.

where *p* and $\varepsilon_{thres}$ are constants to be determined.

**References**

1. Carter, D. R., Fyhrie, D. P. & Whalen, R. T. Trabecular bone density and loading history: regulation of connective tissue biology by mechanical energy. *J. Biomech.* **20,** 785–794 (1987).

2. Wöhler, A. Über die Festigkeitsversuche mit Eisen und Stahl. in *Zeitschrift für Bauwesen* **20,** 73–106 (Ernst & Korn, 1870).

3. *Mechanics of materials*. (McGraw-Hill Education, 2015).
